# The Impact of Financial Literacy on Entrepreneurial Intention: The Mediating Role of Saving Behavior

**DOI:** 10.3389/fpsyg.2022.911605

**Published:** 2022-06-10

**Authors:** Ali Saleh Alshebami, Salem Handhal Al Marri

**Affiliations:** ^1^The Saudi Investment Bank Chair for Investment Awareness Studies, The Deanship of Scientific Research, The Vice Presidency for Graduate Studies and Scientific Research, King Faisal University, Al Ahsa, Saudi Arabia; ^2^Applied College in Abqaiq, King Faisal University, Al Ahsa, Saudi Arabia; ^3^Adam Smith Business School, University of Glasgow, Glasgow, United Kingdom

**Keywords:** awareness, SMEs, Saudi Arabia, entrepreneurship, entrepreneurs

## Abstract

This study explored the impact of financial literacy (financial awareness) on potential entrepreneurs' intent in Saudi Arabia. It also examined saving behavior as a mediator in the relationship between financial literacy and entrepreneurial intention. The study's data were collected by an online questionnaire sent to a sample of 270 potential entrepreneurs at Abqaiq Applied College, affiliated with King Faisal University. Data analysis was done using partial least squares structural equation modeling (PLS-SEM). According to the findings, there is no direct relationship between financial literacy and entrepreneurial intent. However, it has been reported that saving behavior can mediate between financial literacy and entrepreneurial intent.

## Introduction

Entrepreneurship and the small and mid-size enterprise (SME) have been identified as critical drivers of economic growth and development, as well as a means of alleviating poverty (Li et al., 2020a; Yaser Al-Mamary et al., 2020; Alshebami, 2021; Cai et al., 2021; Cheng et al., 2021). Entrepreneurship and SMEs have recently received a lot of attention from various scholars and institutions because of their role in assisting individuals and allowing them to develop new ideas, products and services that result in a higher standard of living. More support, however, is still required for their overall development. This support can be institutional (Ali et al., 2021) or psychological, such as enhancing individuals' personal traits (Bhatti et al., 2021). More specifically, the development of entrepreneurial intent among individuals, particularly potential entrepreneurs (qualified students), would necessitate early preparation. Individuals, for example, must develop high self-confidence, tolerate risk, and be willing to innovate (Koh, 1996; Nasip et al., 2017).

In addition, potential entrepreneurs must educate themselves financially and instill the habit of saving to make better investment decisions, make appropriate planning decisions, and seize available business opportunities in the market (Kilara and Latortue, 2012; Rikwentishe et al., 2015a; Li et al., 2020b). This is because financial education, also known as financial knowledge (Gilenko and Chernova, 2021), financial literacy, or financial awareness, is an essential aspect of individuals' financial wellbeing and financial empowerment (Ali et al., 2021). Indeed, financial literacy is defined as the ability to comprehend and apply fundamental financial principles to effectively allocate financial resources and identify market opportunities (Li and Qian, 2020).

It is also the ability to make sound financial and wealth-generation decisions (Mitchell and Lusardi, 2015). Individuals with a high level of financial literacy can easily develop the necessary risk management skills, identify available business opportunities, gain more market knowledge, manage their money more effectively, and make better financial decisions, all of which are critical for the growth of venture creation and entrepreneurship (Hilgert et al., 2003; Klapper et al., 2015; Li and Qian, 2020; Qader et al., 2022). Furthermore, financial literacy can help individuals develop saving habits (Hilgert et al., 2003; Sabri and MacDonald, 2010), leading to the establishment of new businesses or the expansion of existing ones (Rikwentishe et al., 2015b).

Despite the existence of some literature discussing the concept of financial literacy and saving behavior, it is still believed that there is a dearth of research in this area (Lusardi et al., 2009; Arnida et al., 2015), particularly related to young adult saving and financial literacy in developing countries. As a result, it is critical to investigate how financial literacy and saving habits influence the entrepreneurial behavior of potential entrepreneurs (qualified students) in Saudi Arabia, as it is believed that Saudi Arabia has promising economic indicators related to entrepreneurship and SMEs (Ali et al., 2021).

Furthermore, because Saudi Arabia's state budget is heavily reliant on oil revenue, it has found it challenging to meet its obligations due to the budget deficit caused by the continuous fluctuation in oil prices. As a result, the Saudi government created the so-called Saudi Vision 2030, a long-term plan to implement various reforms in various sectors of the economy, including entrepreneurship and SME sectors. The Saudi Vision 2030 aimed to increase the contribution of the SME sector to GDP from 20 to 35%, increase household savings from 6 to 10% of total household income, and reduce unemployment from 11.6 to 7% (Khan and Alsharif, 2019; Aljarodi, 2020; Alshebami et al., 2020; Elnadi and Gheith, 2021). Consequently, to meet the goals of Saudi Vision 2030, it is critical to investigate the impact of certain factors, such as financial literacy and saving behavior, on the entrepreneurial behavior of potential entrepreneurs. This is because it is believed that financial literacy and saving affect individuals' entrepreneurial intent and business creation, either directly or indirectly (Hilgert et al., 2003; Rikwentishe et al., 2015b; Li and Qian, 2020).

However, despite the importance of financial literacy in enhancing entrepreneurial behavior among individuals, it was discovered that there is still a paucity of literature on the subject (Li and Qian, 2020). Moreover, few studies have focused on saving and its role in business creation (Otto, 2009). Additionally, there is a significant gap in financial literacy levels between developed and developing countries (Lusardi and Mitchell, 2011). According to the World Bank, the Arab world is ranked as one of the least financially literate regions (The World Bank, 2016). Saudi Arabia, in particular, was identified as one of the poorest countries in terms of financial literacy, with men accounting for ~34% and women accounting for ~29% (Hasler and Lusardi, 2017).

Although only about half of the world's adult population saves money regularly (Demirguc-Kunt et al., 2018), Saudi Arabia was identified as a country with a lower rate of savings when compared to other countries (The General Authority for Statistics, 2020). Saudis have a financial literacy rate of around 31% (Alshebami and Seraj, 2021). The low savings rate in Saudi Arabia can be attributed to various factors, including low income (King Khaled Foundation, 2018) and a lack of financial literacy (Hailesellasie et al., 2013). Low financial literacy in Saudi Arabia is more prevalent among young people under 37 years old. Therefore, there is a need to improve financial literacy in that group (Sedais and Al Shahab, 2020).

Accordingly, we argue that financial awareness or literacy is required to develop saving habits and entrepreneurial behavior (Delafrooz and Paim, 2011; Li and Qian, 2020). To put it another way, Those individuals who can develop a high level of financial literacy can obtain essential skills to make sound investments and financial decisions, increase their financial freedom, improve their standard of living, and increase their confidence and autonomy (Sohn et al., 2012; Philippas and Avdoulas, 2019; Ali et al., 2021; Gilenko and Chernova, 2021). Financial literacy also helps to prepare individuals with entrepreneurial financial skills, market knowledge, finance sources, financial knowledge, and entrepreneurial intent (Hilgert et al., 2003; Levesque et al., 2009; Li and Qian, 2020). It also enables them to recognize and capitalize on available business (Evan and Jovanovic, 1989). As a result, the following questions will be addressed in this study:

1. Is there a link between financial literacy and entrepreneurial intent among potential Saudi entrepreneurs?
2. Can saving behavior in Saudi Arabia mediate the relationship between financial literacy and the entrepreneurial intent of potential entrepreneurs?

The article is divided into the following sections. After the introduction, it discusses the extant literature and the development of hypotheses. The work then shifts to discussing the research methodology, analysis of the results, and discussion. It then concludes by presenting the implications and conclusions of the study.

## Literature Review and Hypothesis Development

### Theoretical Underpinnings

According to established theories such as Ajzen (1991) theory of planned behavior and Bandura (1986) social cognitive theory, external factors influence individuals' intentions to perform specific behaviors. These external influences directly influence how people think, perceive and act. In general, intent is a good predictor of individual behavior in the context of business creation. An individual's desire to start their own business is also a cognitive state (Bullough et al., 2014). Personal traits (Koh, 1996; Nasip et al., 2017; Jiatong et al., 2021; Murad et al., 2021; Rafiq and Muhammad, 2021) or level of financial literacy (Evan and Jovanovic, 1989) or individual saving behavior are just a few of the factors that can influence an individual's entrepreneurial intention (Kilara and Latortue, 2012; Rikwentishe et al., 2015b; Tshiaba et al., 2021).

Furthermore, concerning the financial literacy theoretical background, it has been observed that there are no specific measures for measuring financial literacy (Cole and Fernando, 2008; Oseifuah, 2010). We use the measures that measure an individual's knowledge level and behavior developed by Cude et al. (2006) and Thung et al. (2012). Accordingly, we define financial literacy as individuals' ability to make efficient decisions and judgements when managing personal finances (Stolper and Walter, 2017). Also, we use the behavioral life cycle theory (BLCT), which states that mental accounting, framing and self-control are methods for enhancing the savings behavior of individuals (Mpaata et al., 2021).

### Financial Literacy and Entrepreneurial Intention

Financial literacy is defined as people's knowledge of financial concepts and how to apply this knowledge to make sound financial decisions (Stolper and Walter, 2017). Financial literacy raises people's awareness of business opportunities and the necessary risk management skills and market knowledge for developing entrepreneurship and business profit (Hilgert et al., 2003). Financial literacy is essential, especially in light of evidence pointing to a lack of funds as a barrier to creating new ventures (Li and Qian, 2020); financial literacy will notify the entrepreneurs of the necessary financial sources for funding their business. Furthermore, because a lack of financial literacy and awareness leads to higher borrowing costs and more debts (Stango and Zinman, 2009), as well as poor financial behavior and business investment, it is assumed that a high level of financial awareness or literacy will lead to a better understanding of finance and its financial means (Li and Qian, 2020). Financial literacy alerts entrepreneurs to the necessary financial sources for funding their business (Glaser and Walther, 2014).

Moreover, financial literacy specifically assists potential entrepreneurs in making better financial decisions, identifying better sources of funding for their start-ups (Levesque et al., 2009), managing their enterprises' budgets, and making strategic business investment decisions. It also aids in the development of entrepreneurial skills, such as recognizing and capitalizing on available market business opportunities (Evan and Jovanovic, 1989). Individuals with higher financial literacy are likelier to act wisely when making risky business investment decisions (Gilenko and Chernova, 2021). They are likelier to participate in more financial services and products and save and invest (Hogarth and Hilgert, 2002).

According to resource-based theory, financial literacy is an intangible source for the entrepreneurial firm (RBV). This is because a high level of financial literacy among individuals in general, and young people in particular, may contribute to promoting entrepreneurial activities such as autonomy and motivation, self-employment (Oseifuah, 2010; Li and Qian, 2020), as well as facilitating the financing source for them. As a result of the previous discussion, we propose the following hypothesis:

*H1: Financial literacy and entrepreneurial intent have a positive relationship*.

### The Mediating Role of Saving Behavior Between Financial Literacy and Entrepreneurial Intention

Financial literacy is defined as people's ability to clearly understand and interpret their finances to make sound financial decisions. Financial literacy significantly impacts various factors, including individuals' saving behavior (Thung et al., 2012; Arnida et al., 2015). Individuals with a high level of financial literacy can ensure greater financial stability and save more funds for unforeseen events and needs. Individuals' saving behavior is influenced positively by financial literacy (Hilgert et al., 2003; Lusardi et al., 2009; Sabri and MacDonald, 2010; Delafrooz and Paim, 2011). This means that the greater one's financial literacy, the greater one's level of savings, and the greater one's financial wellbeing (Browning and Lusardi, 1996; Gilenko and Chernova, 2021).

Furthermore, although there is very little empirical evidence linking saving and financial literacy (Supanantaroek et al., 2016), existing literature on poor financial literacy indicates that young people with low financial literacy have difficulties managing their economic activities (Sabri et al., 2008), which reduces their ability to save money. As a result, financial literacy encourages people to save regularly, which benefits both individuals and the economy (Gilenko and Chernova, 2021). Individual saving and investment behavior can be influenced by subjective financial assessment as well as personal characteristics (Furnham and Cheng, 2019) as well as personal characteristics.

Furthermore, because financial literacy increases individual savings, it enables people to start new businesses or expand existing ones (Rikwentishe et al., 2015b). Saving provides individuals who want to start small businesses with the necessary business capital and enables them to meet their liquidity challenges (Dunn and Holtz-Eakin, 2000; Kilara and Latortue, 2012; Bosumatari, 2014). Savings are essential for funding individuals and businesses, especially potential entrepreneurs or so-called students (Kilara and Latortue, 2012). As a result, those with good saving habits can quickly develop an entrepreneurial mindset and start making money (Bosumatari, 2014). Saving is thought to be an efficient way of accumulating funds. Therefore, a positive relationship between entrepreneurial development and saving habits develops (Erskine et al., 2006; Rikwentishe et al., 2015b).

Consequently, it is argued that savings can serve as a link between financial literacy and entrepreneurial intent. This is because financial literacy instills in people the necessary skills and perceptions of saving behavior, highlighting the importance of saving in individuals' financial stability and wellbeing, and making sound financial and business decisions. Furthermore, once an individual saves and collects the necessary funds, they can be used in a variety of entrepreneurial investments, such as meeting future challenges and creating new or expanding existing job opportunities (Okeke et al., 2015; Rikwentishe et al., 2015b; Supanantaroek et al., 2016). To conclude, it is argued that people with strong self-control and financial literacy are likelier to save, invest, and develop entrepreneurial intentions. As a result, we propose the following hypothesis:

*H2: Saving behavior mediates the relationships between financial literacy and entrepreneurial intention*.

### Hypothesized Model

**Figure 1:**
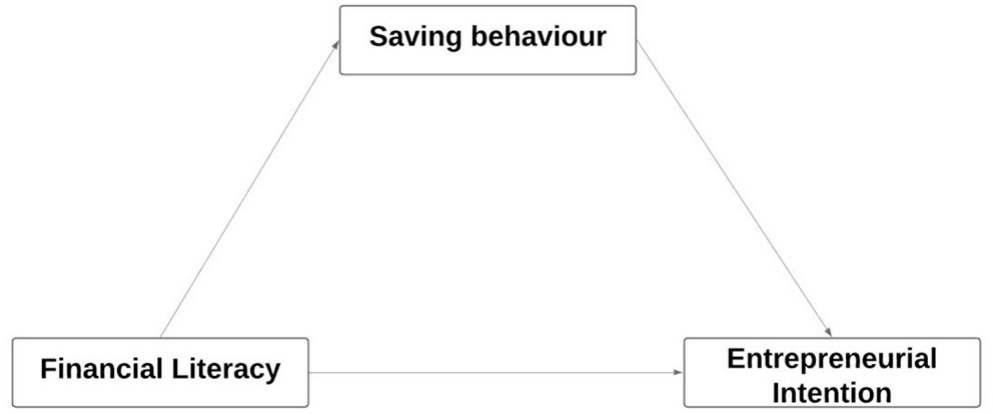
Study model. Source: Authors' elaboration.

In this model, shown in Figure 1, financial literacy is an independent variable, entrepreneurial intention as a dependent variable, and saving behavior is a mediating variable that affects the relationship.

## Research Methodology

### Participants and Procedures

The quantitative study was conducted using a self-administered questionnaire sent to 270 potential entrepreneurs (qualified students) of the Applied College of Abqaiq, affiliated with King Faisal University in Saudi Arabia. The study's target respondents consist of applied college students pursuing two types of programmes: Human Resource Management and Medical Secretary. Compared to other students with bachelor's degrees and other degrees, these students have the most difficulty finding jobs after graduation. As a result, they are expected to establish small entrepreneurial businesses.

The study respondents were chosen because they had studied a subject called “financial management,” which provided them with the necessary information for financial planning. They also learned about various topics and received specific entrepreneurship training. The study included both male and female participants. Similar surveys from previous studies were used to develop the study's questionnaire. The survey questionnaire was distributed to respondents *via* an online link. However, before distribution, a pilot study with 15 respondents was conducted to assess the suitability of the measurers and the questionnaire. Because there were no issues with the questionnaire criteria, the questionnaire link was then sent to respondents and made available online for 1 month.

### Respondents' Demographic Information

Table 1 summarizes the study's 270 respondents, of which 125 were females and 145 were males.

**Table 1:** Respondents' demographic information.

	**Type**	**Percentage**	**Frequency**
Major	Medical secretary	6	17
	Human resource management	94	253
Total		100	270
Gender	Female	46	125
	Male	54	145
Total		100	270

*Source: Primary data*.

As shown in Table 1, the majority of respondents (94%) were from the human resource programme, while only 6% were from the medical secretary programme.

### Sources of Measures Used in the Study

Table 2 shows the sources of measurements used in the study for financial literacy, entrepreneurial intentions, and saving behavior.

**Table 2:** Sources for the study measures.

	**Constructs**	**Source**
1	Financial literacy	Cude et al., 2006; Thung et al., 2012
2	Entrepreneurial intention	Liñán and Chen, 2009
3	Saving behavior	Sabri and MacDonald, 2010; Delafrooz and Paim, 2011

*Source: Author's elaboration*.

The measures of the study were evaluated using a 5-point Likert scale, showing five in total agreement and one in complete disagreement.

## Analysis of Data

Two steps were taken to evaluate the data and interpret the study's findings: (1) examine the measurement model, and (2) examine the structural model.

### Measurement Model Analysis

The purpose of the measurement model was to investigate the convergent validity and reliability of the study items and constructs. Thus, we began by assessing the so-called factor loading of all indicators. To ensure better reliability of the items used in the study, we recommended that the values of each indicator have a loading value of 0.70 or higher to ensure that the measured construct could explain 50% of the variance in the indicator used for analysis, revealing good reliability (Hair et al., 2019). However, it should be noted that even though there might be items below 0.70 loading values, their removal should be based on the condition that their removal will lead to more composite reliability. It should also be noted that items between 60 and 70% loadings were accepted in the exploratory research. Nevertheless, those items with loading values below 0.40 were removed (Hair et al., 2011, 2017).

In the second step of the measurement model, we evaluated the composite reliability used to assess the reliability of the internal consistency of the study constructs. The higher the composite reliability, the greater the reliability. Composite reliability should be between 60 and 70% (Hair et al., 2017). In the third step, we evaluated convergent validity, which assesses how one measure compares favorably to another measure of the same construct. In this case, we examined convergent validity using the extracted average variance (AVE). We used the recommended AVE of 50% or above, as it demonstrates the ability of the construct to explain more than 50% of the variance in the indicator (Hair et al., 2011, 2019).

Table 3 contains a description of the reliability and convergent validity. It shows that after removing the unwanted indicators, the results are in line with the recommended values, indicating the presence of adequate reliability and validity.

**Table 3:** Reliability and convergent validity.

**Construct**	**Loadings**	**Composite reliability**	**Average variance extracted (AVE)**
Entrepreneurial intention		0.892	0.581
E1	0.666		
E2	0.810		
E3	0.752		
E4	0.793		
E5	0.784		
E6	0.760		
Financial literacy		0.847	0.527
FL1	0.793		
FL2	0.792		
FL3	0.641		
FL4	0.739		
FL7	0.650		
Saving behavior		0.864	0.515
SB1	0.688		
SB4	0.759		
SB5	0.740		
SB6	0.724		
SB7	0.682		
SB8	0.708		

*Source: Primary data*.

After completing step 3, we proceeded to step 4 of the measurement model, which determines the discriminant validity of the study constructs. This step illustrated how distinctive one construct was compared to the other constructs in the structural model (Hair et al., 2019).

To assess the discriminate validity, the Heterotrait-Monotrait Ratio (HTMT) was used. Table 4 shows how empirically distinct one construct in the structural model is from the others. It also assumes that the sum of all model construct variances cannot be greater than their individual variances. The HTMT values did not exceed 0.90, indicating that the study constructs had adequate discriminate validity (Henseler et al., 2015).

**Table 4:** Heterotrait–Monotrait ratio (HTMT).

	**Entrepreneurial intention**	**Financial literacy**	**Saving behavior**
Entrepreneurial intention	0.762		
Financial literacy	0.464	0.726	
Saving behavior	0.664	0.545	0.717

*Source: Primary data*.

### Analysis of the Structural Model

#### Collinearity Issue

After examining various tests in the measurement model, we evaluated the first step in the structural model. To ensure that the regression results were free of bias, we began by investigating the collinearity issue. As a result, we put the so-called variance inflation factor to the test (VIF). Thus, if the VIF value was >5, the study would be deemed to have collinearity issues.

Table 5 shows that all of the reported values are <3, indicating no collinearity.

**Table 5:** Collinearity results.

	**Entrepreneurial intention**	**Saving behavior**
**Entrepreneurial Intention**		
Financial literacy	1.422	1.000
Saving	1.422	

#### Explanatory Power

After examining the collinearity, we reviewed the coefficient of determination (*R*^2^), also known as the model's explanatory power. This test was carried out by calculating *R*^2^, the sum of the independent variables' effects on the dependent variables.

The model's explanatory power was deemed adequate because the *R*^2^ in Table 6 was more significant than 0.25. In other words, the model could account for ~45% of the variance in entrepreneurial intention. There is no rule of thumb for *R*^2^ because the outcome can vary based on the field of study and context.

**Table 6:** Coefficient of determination (*R*^2^).

	***R* square**	***R* square adjusted**
Entrepreneurial Intention	0.456	0.452
Saving behavior	0.297	0.294

*Source: Primary data*.

#### Construct Cross-Validated Redundancy

In Table 7, we see the constructs' cross-validated redundancy results. The 1-SSE/SSO values were higher than zero. Accordingly, the study's model had adequate predictive power.

**Table 7:** Construct cross-validated redundancy.

	**SSO**	**SSE**	***Q*^2^ (= 1–SSE/SSO)**
Entrepreneurial intention	1,620.000	1,199.585	0.260
Financial literacy	1,350.000	1,350.000	
Saving behavior	1,620.000	1,387.700	0.143

*Source: Primary data*.

#### Hypothesis Testing and Results

This section is important because it describes the bootstrapping procedure used to test the hypotheses with 500 resamples.

Table 8 shows the relationship between the variables in the study. Accordingly, hypothesis H1 is rejected because it demonstrates no direct positive relationship between financial literacy and entrepreneurial intention (β = 0.145, *P* > 0.05). However, when saving behavior was included as a mediator between financial literacy and entrepreneurial intention, the findings revealed that saving behavior could fully mediate the previously mentioned relationships. As a result, H2 was accepted (β = 0.319, *P* < 0.05).

**Table 8:** Path coefficients.

		**β**	***M***	***t*-value**	***p*-value**	**Decision**
H1	Financial literacy → Entrepreneurial intention	0.145	0.143	1.880	0.061	Rejected
H2	Financial literacy → Saving behavior → Entrepreneurial intention	0.319	0.326	6.872	0.000	Accepted

*Source: Primary data*.

#### Path Coefficient Representations

**Figure 2:**
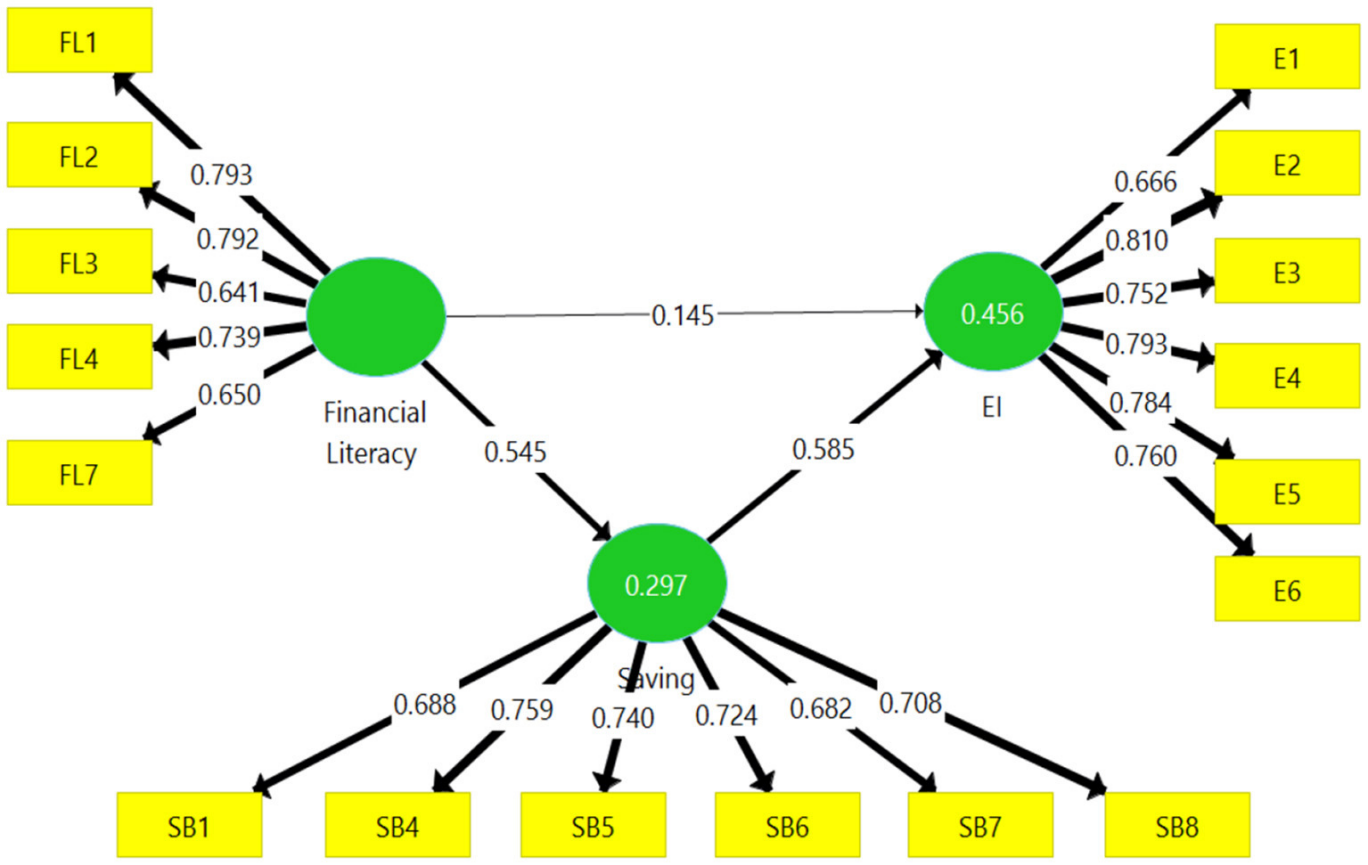
Path coefficient results. Source: Primary data.

Figure 2 demonstrates the path coefficients of the study's different constructs.

## Discussion

This study investigated how the financial literacy of potential entrepreneurs (qualified students) influences their entrepreneurial behavior and business establishment decisions. The study also aimed to examine how potential entrepreneurs' saving behavior can mediate the relationship between their level of financial literacy and their entrepreneurial intention. As a result of investigating the hypothesized relationships of the study constructs, intriguing results were discovered. It was first reported that financial literacy among potential entrepreneurs does not always result in the development of entrepreneurial intentions.

This finding was surprising because it was revealed otherwise in most previous literature. However, this result could be attributed to the fact that, while those potential entrepreneurs may have the intention to start their small entrepreneurial firms, they may be hampered by other environmental factors, such as formal and informal institutions or a lack of necessary financial support, leading them to abandon the idea of starting a venture. This may confirm the need for financial support and the need to instill a saving culture in individuals' mindsets. This finding could also be attributed to the fact that those potential entrepreneurs have a limited understanding of financial literacy, which is insufficient to change their mindsets and direct their behavior toward business creation. This finding is similar to those of Ojogbo et al. (2022), who reported no connection and revealed a negative association between financial literacy and entrepreneurial intention. Ojogbo is also not consistent with previous studies confirming a positive relationship between financial literacy and entrepreneurial intent, such as Hilgert et al. (2003) and Levesque et al. (2009).

Hypothesis H2, which stated that saving behavior mediates the relationship between financial literacy and entrepreneurial intention, was accepted. Acceptance was expected because as more people develop financial knowledge, literacy, or awareness, they become more knowledgeable about financial issues, save money, manage their personal finances wisely, and take advantage of the market's available finance and entrepreneurial opportunities (Delafrooz and Paim, 2011). They also recognize the significance of saving behavior as a source of finance for individuals. As a result, they begin saving and reinvesting in future investments and entrepreneurial firms (Arnida et al., 2015). Saving is considered vital because it connects individuals' financial literacy, entrepreneurial intent, and business creation. It also enables them to face future financial challenges, uncertainties and potential risks. Saving also allows them to reduce their reliance on external sources of finance, which are often inaccessible and expensive. This research supports the findings of previous studies (Hilgert et al., 2003; Sabri and MacDonald, 2010; Delafrooz and Paim, 2011; Thung et al., 2012; Arnida et al., 2015), which emphasize the importance of financial literacy and saving in the development of entrepreneurial intention and behavior.

## Implications

The continuous rise in the unemployment rate and other socioeconomic problems in various parts of the world, particularly in countries with oil-based economies, has compelled them to devise new strategies for dealing with these challenges. Consequently, Saudi Arabia, as one of the oil-producing countries affected by these events, developed its own strategy, including the so-called Saudi Vision 2030, to address these challenges, particularly to support SMEs and entrepreneurship (Alshebami et al., 2020; Ali et al., 2021; Elnadi and Gheith, 2021). Accordingly, this article contributes new literature to the existing literature on the impact of financial literacy and saving behavior in supporting SMEs and potential entrepreneurs' entrepreneurial intentions. It also provides empirical evidence regarding the effects of financial literacy on entrepreneurial intention and the mediation effect of saving behavior on entrepreneurial intention among potential entrepreneurs.

Furthermore, the study emphasizes the need to broaden the study's scope and sample size to allow researchers to examine the impact of financial literacy and other financial concepts from various perspectives. The study also emphasizes the importance of improving financial literacy and instilling saving habits in potential entrepreneurs to maximize the benefits of their entrepreneurial firms' creation (Dunn and Holtz-Eakin, 2000; Kilara and Latortue, 2012; Rikwentishe et al., 2015a). It also highlights that good saving habits can be a good source of start-up capital (Dozie, 1995; Bosumatari, 2014).

The study recommends that policymakers, educational institutions, and other development programmes focus on developing the necessary financial literacy programmes and saving curricula and include them as an essential part of their university syllabus to maximize the benefits of potential entrepreneurs and direct them toward starting their small businesses. While universities and other educational institutions develop the necessary financial literacy programmes, banks and other financial institutions must work on creating different types of saving services to encourage individuals to save. Furthermore, financial institutions and banks should provide more support and financial grants to scholars to continue researching other aspects of financial literacy and investment awareness and their impact on the SME and entrepreneurship sectors. Finally, policymakers in Saudi Arabia must popularize the concept of financial awareness or literacy and direct it toward supporting the development of entrepreneurship.

## Conclusions

The significance of instilling entrepreneurial intention and behavior in potential entrepreneurs necessitated identifying key factors that may contribute to the development of potential entrepreneurs' entrepreneurial behavior. As a result, we investigated how financial literacy and saving behavior can influence potential entrepreneurs' entrepreneurial intentions (qualified students). The study sought to examine the perceptions of 270 potential entrepreneurs at the applied college of Abqaiq, affiliated with King Faisal University, regarding financial literacy, saving behavior and entrepreneurial intention. The study found an interesting relationship between financial literacy and entrepreneurial intention and the ability of saving behavior to mediate the relationship between financial literacy and entrepreneurial intention in the study context. We also concluded that the greater individuals' financial literacy, the greater their potential savings. Furthermore, the more one saves, the more likely to engage in entrepreneurial behavior.

As a result, policymakers and the private sector must focus on and collaborate on increasing financial literacy and saving behavior among potential entrepreneurs. This behavior can be accomplished by growing educational programmes on financial literacy and developing more saving products and services to encourage potential entrepreneurs. The entrepreneurs, in turn, would become more involved in them and as a result, develop a propensity for business creation. Finally, even though this study contains some intriguing findings, it is essential to note that it has some limitations regarding its sample and the concepts used. Because of the small sample size, it is difficult to generalize the findings. Consequently, it is suggested that future studies include larger sample sizes, more concepts on the regression process, control variables, and broaden the context of the study by comparing other countries to Saudi Arabia.

## Data Availability Statement

The raw data supporting the conclusions of this article will be made available by the authors, without undue reservation.

## Ethics Statement

The studies involving human participants were reviewed and approved by King Faisal University. Written informed consent for participation was not required for this study in accordance with the national legislation and the institutional requirements.

## Author Contributions

Both authors listed have made a substantial, direct, and intellectual contribution to the work and approved it for publication.

## Funding

This work was supported by the Saudi Investment Bank Chair for Investment Awareness Studies, the Deanship of Scientific Research, the Vice Presidency for Graduate Studies and Scientific Research, King Faisal University, Saudi Arabia [Grant No. 32].

## Conflict of Interest

The authors declare that the research was conducted in the absence of any commercial or financial relationships that could be construed as a potential conflict of interest.

## Publisher's Note

All claims expressed in this article are solely those of the authors and do not necessarily represent those of their affiliated organizations, or those of the publisher, the editors and the reviewers. Any product that may be evaluated in this article, or claim that may be made by its manufacturer, is not guaranteed or endorsed by the publisher.
